# Physiology of Grafting

**Published:** 1899-10

**Authors:** 


PHYSIOLOGY OF GRAFTING.—M. L. Daniel distinguishes two stages in the anatomical processes which take place in grafting herbaceous or woody plants—the preliminary and the definitive stages. The former stage extends up to the moment when the functions of the outer and inner generating layers, interrupted by the operation of grafting, are resumed. The definitive stage may again be divided into two periods—the formation of the cellular tissues which fill up the vacancies caused by the wound, and the differentiation of the vessels and sieve-tubes in the healing tissues produced by the activity of the inner generating layer, and the formation of bark and phelloderm.—*Bonnier's Rev. Gen. de Bot.*, 11, 78.

